# The Axial Length of the Eyeball and Bioelectrical Activity of Masticatory and Neck Muscles: A Preliminary Report

**DOI:** 10.1155/2022/6115782

**Published:** 2022-08-16

**Authors:** Grzegorz Zieliński, Michał Baszczowski, Maria Rapa, Anna Matysik-Woźniak, Magdalena Zawadka, Jacek Szkutnik, Piotr Gawda, Robert Rejdak, Piotr Majcher, Michał Ginszt

**Affiliations:** ^1^Department of Sports Medicine, Medical University of Lublin, Lublin, Poland; ^2^Interdisciplinary Scientific Group of Sports Medicine, Department of Sports Medicine, Medical University of Lublin, Lublin, Poland; ^3^Students' Scientific Association at the Department of General and Pediatric Ophthalmology, Medical University of Lublin, Lublin, Poland; ^4^Department of General and Pediatric Ophthalmology, Medical University of Lublin, Lublin, Poland; ^5^Independent Unit of Functional Masticatory Disorders, Medical University of Lublin, Lublin, Poland; ^6^Department of Rehabilitation and Physiotherapy, Medical University of Lublin, Lublin, Poland

## Abstract

**Objective:**

The present study aimed to evaluate the correlation of eye length and bioelectric activity of temporalis, masseter, digastric, and sternocleidomastoid muscles in women with myopia compared to healthy women.

**Methods:**

Based on the exclusion and inclusion criteria, 42 women aged 24 years (±2 years) were eligible for the study. Two equally sized groups with myopic (*n* = 21) and emmetropic healthy subjects (*n* = 21) were formed. An electromyographic study of the examined muscles was performed in four conditions: at rest, during maximal voluntary clenching in the intercuspal position, during maximal voluntary clenching on dental cotton rollers, and during maximal mouth opening using BioEMG III (BioResearch Associates, Inc. Milwaukee, WI, USA). The IOL Master 500 (Carl Zeiss Meditec, Jena, Germany) was used to examine the eyeball length. Statistical analysis showed significant positive correlations during mouth opening in both groups with open and closed eyes.

**Results:**

A greater number of correlations between the analyzed variables was observed in emmetropic women. In almost all cases, the longer axial eye length was associated with an increase in the bioelectrical activity of the analyzed muscles. Significant correlations were most often observed within the masseter and digastric muscles during the maximum mouth opening and at rest.

**Conclusion:**

There is a relationship between the bioelectrical activity of the masticatory muscles and the axial length of the eyeball on the same side.

## 1. Introduction

Myopia is a prevalent condition that typically starts in childhood and early adulthood and is characterized by a blurring of objects viewed at a distance [1]. Globally, myopia is estimated at 42.9% in people aged 43–52 years and up to 14.4% in those aged 75 years and older [2]. In Poland, myopia affects between 30.6% and 52% of the adult population [3, 4].

It is predictable that by 2050, there will be over 4750 million people with myopia [5]. This condition is also the most frequent cause of irreversible refractive error in the working population [6, 7]. The etiology of myopia is multifactorial and not fully understood. Evidence suggests that myopia is a consequence of the interaction between genetic and environmental components [8]. Myopia is connected with numerous ocular complications, such as retinal detachment, cataract, glaucoma, optic nerve disc changes, and maculopathy [9]. Moreover, the age of onset and myopia progression are the most important predictors of high myopia in later youth [10].

**Figure 1:**
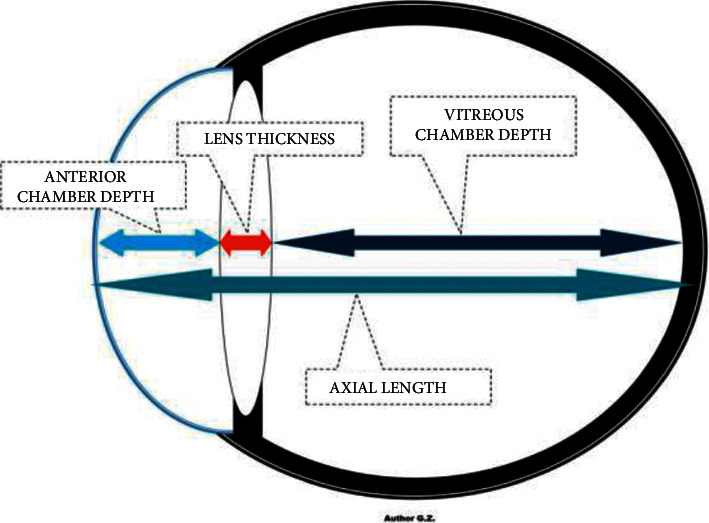
The axial length of the eyeball.

The refractive state of a human eye consists of 4 ocular structures, including the cornea, aqueous fluid, lens, and vitreous [11]. The refractive error results from a mismatch between different optical components of the eye, one of which is the length of the eyeball. The most common type of myopia is axial myopia, which results from the excessive elongation of the eyeball. The eyeball length increases during childhood and adolescence, leading to myopia if this increase in eyeball axial length exceeds the eye's focus [12]. The eyeball length parameters are important data in diagnosing refractive errors in nearsighted (the eyeball is elongated) and farsighted (the eyeball is shortened) patients. The measurements of the parts of the eye, namely the anterior chamber depth, lens thickness, vitreous chamber depth, and axial length, are widely evaluated in ocular diseases (Figure 1). The eyeball length is 24 mm for low myopia (−6 D < refractive error <0 D), whereas the eyeball length for high myopia is approximately 30 mm (refractive error < −6 D) [11].

Several studies suggest the effect of visual impairment on electromyographic activity within the masticatory muscles in myopic subjects [13–16]. As we previously reported, closing and opening eyes may be related to changes within the bioelectric activity of the cervical and masticatory muscles in myopic patients [16]. Increased bioelectric activity and reorganization of electromyographic patterns within the masticatory muscles may be associated with a predisposition to temporomandibular disorders (TMDs) [17]. Moreover, people with refractive conditions are more affected by headaches than healthy subjects, which may be related to increased bioelectric activity within the anterior temporalis muscles [18, 19]. However, no studies have evaluated the correlation between the axial length of the eyeball and the electromyographic activity within masticatory muscles. Therefore, this study aimed to analyze the correlation between the axial length of the eyeball and bioelectric activity of temporalis, masseter, digastric, and sternocleidomastoid muscles in women with myopia in comparison to healthy women.

## 2. Materials and Methods

### 2.1. Study Population

One hundred and one women were invited to participate in the study. The Bioethics Committee of the Medical University of Lublin approved the presented research (KE-0254/229/2020). The study was conducted following the current principles of the Declaration of Helsinki (64th WMA General Assembly, Fortaleza, Brazil, October 2013). Participants were informed about the study's objectives during the recruitment procedure, and written consent was obtained from all subjects involved.

The inclusion criteria applied in the presented research were as follows: female gender, no visual impairment (control group), myopia based on clinical examination (myopia group), four zones of arch support, and complete dentition.

The following exclusion criteria were applied in the research: hyperopia, eye diseases, optic nerve diseases, TMDs based on the research diagnostic criteria for temporomandibular disorders (RDC/TMD), II and III class according to Angle's classification, oral inflammation, open bite, crossbite, neurological disorders in the head and neck, neoplastic diseases, head, neck, and upper limb pain of any etiology within the last six months, trauma and previous surgical treatment in the head and neck region within the last six months, and pregnancy.

Based on the exclusion and inclusion criteria, 42 women aged 24 years (±2 years) were eligible for the study. Two equally sized groups with myopic *n* = 21 and emmetropic subjects *n* = 21 were formed. The intraocular pressure in the refractive error subjects was 17 mm·Hg (±4 mm·Hg) in the right eye and 14 mm·Hg (±4 mm·Hg) in the left eye. Intraocular pressure in the emmetropic subjects was 17 mm·Hg (±3 mm·Hg) in the right eye and 14 mm·Hg (±4 mm·Hg) in the left eye (Tonopen, Reichert, Depew, New York, USA). The groups did not statistically differ in intraocular pressure between each other (*p* > 0.05).

It was decided to study women because of the more frequent occurrence of myopia [20, 21] and more frequent TMD [22] in comparison to men.

### 2.2. Study Protocol

#### 2.2.1. Ophthalmic Examination

The Snellen chart was used to test visual acuity. The Snellen chart remains the most widely used method for visual acuity testing [23]. It was, therefore, selected for this study. The Snellen chart uses a geometric scale to measure visual acuity, with correction vision at a distance set to 20/20. In the emmetropic group, visual acuity was 20/20. In the refractive error group tested under correction, visual acuity was 20/20.

According to the existing clinical and epidemiologic studies standards, people with myopia were defined as those with a refractive error ≤−0.50 diopters (D) [24]. The myopia group included women with a refractive error of −0.50 D to −5.75 D. The mean refractive error value was −2.5 D (±−1.00 D) for the right eye and −2.5 D (±−1.5 D) for the left eye. No visual impairment was found in the emmetropic group.

**Figure 2:**
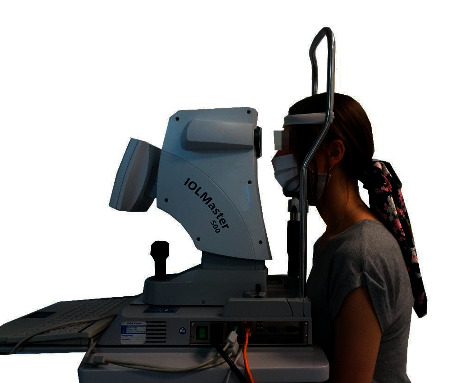
The axial length eyeball measurement during the study.

An IOL Master 500 (Carl Zeiss Meditec, Jena, Germany) was used to examine the eyeball length. This device is precise and is used in ophthalmology to calculate the power of artificial intraocular lenses. It is a noninvasive optical biometer that uses 780 nm partial coherence interferometry to measure the eye's axial length. The axis data are obtained from the optical path distance from the anterior corneal surface to the retinal pigment epithelium [25]. All ophthalmic tests were performed by the same researcher [26]. Participants sat in front of the head of the device, resting their chin and forehead against the tripod (Figure 2). The eyes were focused when the apparatus head was approximately 5.5 cm from the patient. Participants were asked to perform a full blink before the examination to spread an optically smooth tear film over the cornea. Five separate tests were performed to assess the average axial length [27].

#### 2.2.2. Electromyographic Examination

Surface electromyographic examinations (sEMG) were performed between 8 and 12 a.m. The sEMG tests were conducted in the dental chair. The subjects assumed a perpendicular position, with the head resting on the armchair headrest and the lower limbs positioned horizontally and parallelly.

**Figure 3:**
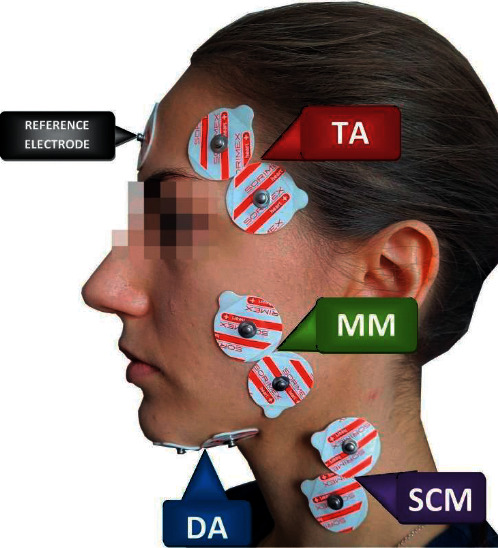
Electrodes placement during the electromyographic examination. TA: temporalis muscle (the anterior part), MM: the masseter muscle (the superficial part), DA: the digastric muscle (the anterior belly), and SCM: the sternocleidomastoid muscle (the middle part).

Before placing surface electrodes, the skin was cleansed with 90% alcohol solution. sEMG electrodes (Ag/AgCl) with a conductive surface of 16 mm (SORIMEX, Torun, Poland) were placed bilaterally on the skin, covering the examined muscle groups. Four pairs of masticatory and neck muscles were analyzed: temporalis muscle (the anterior part-TA), the masseter muscle (the superficial part-MM), the digastric muscle (the anterior belly-DA), and the sternocleidomastoid muscle (the middle part-SCM), according to the SENIAM (surface EMG for noninvasive assessment of muscles) standards and our previous work [28, 29]. The reference sEMG electrode was put on the forehead in the middle of the frontal bone [30] (Figure 3).

Bioelectric muscle activity was measured during the resting mandibular position (10 seconds), maximal voluntary clenching in the intercuspal position (as hard as possible; 3 × 3 seconds, 2 seconds break), maximal voluntary clenching on dental cotton rollers (as hard as possible; 3 × 3 seconds, 2 seconds break), and maximal mouth opening (as wide as possible; 3 × 3 seconds, 2 seconds break). The averaged results from three measurements were used in the statistical calculations [16, 29]. An open eye and closed-eye test were conducted with a 5-minute break between tests. A random choice of the pretest was made. sEMG measurements were performed without visual correction [13, 16].

The reproducibility of the sEMG examination was confirmed by repeated sEMG tests on 10 participants. Two independent sEMG measurements were separated by 5 minutes of rest between the above-mentioned masticatory activities. There were no significant differences (*p* > 0.05) between repeated sEMG results at rest, during maximal voluntary clenching in the intercuspal position, during maximal voluntary clenching on dental cotton rollers, and during maximal mouth opening [3].

The sEMG signal was processed using the BioPAK Measurement System software (BioResearch Associates, Inc. Milwaukee, WI, USA). The microvolt sEMG potentials were amplified with minimal noise to 5000 times their original level. The sEMG noise was reduced by 40 dB using the digital Noise Buster BioPAK measurement system. The electromyography signal processing based on root means square (RMS) calculations produced average bioelectrical values, which were then used for statistical analyses [29]. The strengthening the reporting of observational studies in epidemiology (STROBE) checklist was used to assess the procedural quality of the research [31].

#### 2.2.3. Statistical Analysis

The statistical analysis was performed using the Statistica™ 14.0 (TIBCO Software Inc., Palo Alto, CA, USA). The normal distribution of the data was verified with the Shapiro-Wilk test. The Wilcoxon matched-pairs test for paired samples was used for data, which showed no compatibility with normal distribution. To compare groups, the Mann–Whitney *U* test and *T*-test were used. We compared muscle activity and the axial eye length on the same side in the statistical analysis. The Spearman rank correlation coefficient (R, rho) was used to test the relationship between the axial eye length and the electromyographic activity of the selected muscles. Spearman rho varies between −1 (perfect negative monotonic association) and +1 (perfect positive monotonic association). A correlation was considered large for results greater than 0.5 and moderate for results between 0.3 and 0.5 [32]. Effect sizes were determined for the *t*-test using the Cohen *d* method as small (0.2), medium (0.5), and large (0.8). Statistical significance was set at *p* ≤ 0.05.

## 3. Results

Statistical analysis showed no significant differences between subjects' ages (Table 1) and mandibular mobility ranges (Table 2). Statistically significant differences were shown in the right and left eye axial length between groups (Table 2).

The statistical analysis showed a significant positive correlation between the left axial length and left-sided digastric muscle activity during clenching in the intercuspal position and clenching on dental cotton rollers, both in open and closed eye measurements. The significance of the correlation increased with eyes closed (Table 3).

The statistical analysis revealed a significant positive correlation between axial length and MM-L, DA-L, MM-R, DA-R muscles during maximum mouth opening within both open and closed eye measurements. The significance of the correlation increased with eyes closed (Table 4). In addition, a significant positive correlation was observed during maximum mouth opening within SCM-L muscles (open eyes measurement) and within TA-R (closed eyes measurement). The statistical analysis showed a significant negative correlation within DA-R during rest and during open eyes measurement, as presented in Table 4.

## 4. Discussion

The present study investigated the correlation between the axial length of the eyeball and the bioelectric activity of the masticatory and neck muscles in women with myopia compared to healthy women. In the myopic group, two positive correlations were found between the left axial length and left-sided digastric muscle activity while clenching in the intercuspal position and while clenching on dental cotton rollers, both in open and closed eye measurements. Moreover, the significance of the correlation increased with the eyes closed. Surprisingly, we observed more correlations between the analyzed variables in healthy women without visual impairment. In almost all cases, the longer axial eye parameter was associated with an increase in the bioelectrical activity of the analyzed muscles. Significant correlations were most often observed within the masseter and digastric muscles during the maximum mouth opening and at rest. Therefore, it can be assumed that the activity of these muscles is related to the axial length of the eyeball on the same side, which is most manifested during mandibular abduction and at rest.

An increase in correlation strength during closed eyes measurement may indicate the suppression of the vestibulo-ocular reflex (VOR) [33]. VOR stabilizes retinal image, keeping the eye fixed in space and focused on the object [33, 34]. The information along the VOR pathway is processed by three sources: the visual system through the eyes, proprioceptive receptors, and vestibular receptors in the inner ear. For the brain to perceive the activity of the vestibular system, it must receive information via the afferent pathway from different parts of the vestibular structure [35]. Closing eyes may decrease both VOR and masticatory muscle activity [33].

The hypothetical connection for the presented relationship may be as follows: instead of neurological connections, biomechanical connections in the form of a muscle-fascial network will be important [33]. The link between the organ of vision and the masticatory muscles occurs through the Tenon's fascia, connecting with the deep fascia of the skull, and through it, with the temporal subseries and the deep fascia of the neck [36, 37]. The temporal fascia is a dense fibrous layer covering the temporalis muscle. Its surface provides an attachment site for the superficial fibers of the temporalis muscle. Through the deep cervical fascia, it connects to the quadrilateral and sternoclavicular muscles [36, 37].

In contrast, Tenon's fascia surrounds the eyeball from the edge of the ciliary body to the optic nerve entrance. In the middle part, it attaches to the back of the conjunctiva of the eye. It connects with the eye muscles and the fatty lining of the orbit and forms continuity with the sheath of the optic nerve [36]. The presented correlations were evident, especially while opening the mouth when the fascial network is stretched the most.

The greater number of correlations in healthy/emmetropic versus myopic subjects may be explained precisely by the hypothesized changes associated with it in the fascial network. People with nearsightedness are likely to have changed in the fascial network related to the reduced mobility of the eyeball-glasses limit the field of vision. Hypothetically, a longer knob also interferes with a fascial glide through decreased mobility [33]. All this can lead to fascial disorders called the densification of fascia [38]. It is a thickening of loose connective tissue and its extracellular matrix corresponding to a reduction or loss of gliding ability of the fascia [39]. If loose connective tissue is lost or its density is altered, the behavior of the fascia and underlying muscles is disrupted. The function of the entire muscle-tendon chain is also disrupted [40]. The alteration of the fascial glide can affect mandibular mobility and ocular motility [41]. Hypothetically, changes in the fascia structure can also affect the eye structures, leading, for example, to changes in the sclera observed in axial myopia [42]. The absence of the discussed changes in healthy subjects may explain the higher number of obtained correlations.

Our results revealed a relationship between the bioelectrical activity of the masticatory muscles and the axial length of the eyeball on the same side. In our opinion, the presented phenomenon is related to the biomechanical parameters of the Tenon's fascia. The assessment of these relationships may contribute to a better understanding of the coexistence of dysfunctions within the stomatognathic system and the organ of vision. Further research is necessary to clarify the mechanisms of these connections. The limitation of this study concerned a homogeneous research group. Therefore, it would be desirable to compare the results with the male population in future studies.

## 5. Conclusions

There is a relationship between the bioelectrical activity of the masticatory muscles and the axial length of the eyeball on the same side. Further studies are required to clarify the mechanism of the presented phenomenon.

## Figures and Tables

**Table 1:** Comparison of age between groups.

Variable	Group	Mean	95% CI	95% CI	Median	Minimum	Maximum	SD	*z*	*p*
Age (year)	Myopia	23.52	22.60	24.45	24.00	20.00	30.00	2.04	0.18	0.86
	Control	23.76	22.49	25.03	23.00	19.00	31.00	2.79		

**Table 2:** Comparison of eye axial length and mandibular range of motion between groups.

	Myopia group							Control group								
	Mean	95% CI	95% CI	Median	Minimum	Maximum	SD	Mean	95% CI	95% CI	Median	Minimum	Maximum	SD	*Z*/*t*	*p*
Eye axial length (right eye)	24.46	24.16	24.75	24.47	23.23	25.49	0.65	23.53	23.26	23.80	23.47	22.74	24.79	0.59	*T* = 4.84	**<0.001 ES** **=** **1.50**^*∗*^
Eye axial length (left eye)	24.49	24.14	24.85	24.58	23.04	26.08	0.78	23.53	23.26	23.80	23.49	22.74	24.73	0.59	*T* = 4.50	**<0.001 ES** **=** **1.40**^*∗*^
Active maximum mouth opening	48.05	45.35	50.75	48.00	35.00	59.00	5.76	48.86	46.77	50.94	48.00	42.00	58.00	4.59	*T* = −0.50	0.62
Passive maximum mouth opening	51.00	48.42	53.58	51.50	42.00	62.00	5.51	51.62	49.62	53.62	50.00	46.00	60.00	4.39	*T* = −0.40	0.69
Right lateral movement	10.00	9.32	10.68	10.00	8.00	12.00	1.45	9.52	8.23	10.82	10.00	0.00	13.00	2.84	*Z* = 0.20	0.84
Left lateral movement	10.10	9.17	11.03	10.50	6.00	14.00	2.00	9.71	8.39	11.03	10.00	0.00	14.00	2.90	*Z* = 0.36	0.72
Protrusion	8.80	7.70	9.90	9.50	4.00	13.00	2.35	8.90	7.72	10.09	9.00	3.00	13.00	2.61	*T* = −0.13	0.89

**Table 3:** Pooled correlation results in subjects with myopia.

			Eyes open			Eyes closed		
			Spearman	*t* (*N*-2)	*p* value	Spearman	*t* (*N*-2)	*p* value
			*R*			*R*		
		Myopia group						
Left axial length	Rest	TA-L	−0.14	−0.59	0.56	0.03	0.12	0.90
		MM-L	−0.14	−0.62	0.54	−0.15	−0.66	0.52
		SCM-L	−0.08	−0.33	0.74	0.07	0.33	0.75
		DA-L	0.13	0.55	0.59	0.04	0.18	0.86
	Clenching in the intercuspal position	TA-L	0.19	0.85	0.40	−0.01	−0.05	0.96
		MM-L	0.22	1.01	0.33	0.21	0.93	0.37
		SCM-L	0.25	1.12	0.28	0.26	1.18	0.25
		DA-L	0.56	2.98	**0.01** ^*∗*^	0.62	3.45	**<0.001** ^*∗*^
	Clenching on dental cotton rollers	TA-L	0.22	0.98	0.34	0.18	0.79	0.44
		MM-L	0.18	0.80	0.43	0.14	0.60	0.56
		SCM-L	0.22	1.00	0.33	0.18	0.80	0.43
		DA-L	0.48	2.35	**0.03** ^*∗*^	0.50	2.53	**0.02** ^*∗*^
	Maximum mouth opening	TA-L	−0.22	−1.01	0.33	−0.12	−0.51	0.61
		MM-L	−0.12	−0.51	0.61	−0.11	−0.48	0.64
		SCM-L	0.00	−0.01	0.99	0.02	0.09	0.93
		DA-L	−0.05	−0.23	0.82	−0.13	−0.57	0.57

Right axial length	Rest	TA-R	−0.24	−1.06	0.30	−0.26	−1.15	0.26
		MM-R	−0.07	−0.29	0.78	0.06	0.25	0.81
		SCM-R	0.07	0.32	0.76	−0.01	−0.03	0.98
		DA-R	−0.06	−0.28	0.78	−0.13	−0.56	0.58
	Clenching in the intercuspal position	TA-R	0.15	0.67	0.51	0.22	0.99	0.33
		MM-R	0.25	1.11	0.28	0.23	1.03	0.32
		SCM-R	0.02	0.11	0.92	0.07	0.31	0.76
		DA-R	0.21	0.91	0.37	0.30	1.35	0.19
	Clenching on dental cotton rollers	TA-R	0.24	1.06	0.30	0.22	0.99	0.33
		MM-R	0.25	1.13	0.27	0.42	2.03	0.06
		SCM-R	0.03	0.14	0.89	0.03	0.13	0.90
		DA-R	−0.01	−0.03	0.98	0.22	0.97	0.35
	Maximum mouth opening	TA-R	−0.32	−1.46	0.16	−0.25	−1.11	0.28
		MM-R	−0.29	−1.32	0.20	−0.30	−1.36	0.19
		SCM-R	−0.02	−0.10	0.92	−0.01	−0.06	0.95
		DA-R	−0.25	−1.12	0.28	−0.27	−1.23	0.23

**Table 4:** Pooled correlation results in healthy subjects.

			Eyes open			Eyes closed		
			Spearman	*t* (*N*-2)	*p* value	Spearman	*t* (*N*-2)	*p* value
			*R*			*R*		
		Control group						
Left axial length	Rest	TA-L	−0.11	−0.48	0.64	0.07	0.31	0.76
		MM-L	−0.22	−0.99	0.34	0.05	0.24	0.81
		SCM-L	−0.09	−0.40	0.69	0.26	1.19	0.25
		DA-L	−0.33	−1.52	0.14	−0.01	−0.04	0.97
	Clenching in the intercuspal position	TA-L	0.40	1.92	0.07	0.36	1.69	0.11
		MM-L	0.16	0.71	0.49	0.19	0.83	0.42
		SCM-L	0.14	0.60	0.56	0.19	0.83	0.41
		DA-L	−0.14	−0.63	0.54	−0.15	−0.67	0.51
	Clenching on	TA-L	0.08	0.34	0.74	−0.06	−0.26	0.80
	Dental cotton rollers	MM-L	0.20	0.87	0.39	0.09	0.39	0.70
		SCM-L	0.08	0.36	0.72	0.00	0.00	0.99
		DA-L	−0.01	−0.05	0.96	−0.16	−0.69	0.50
	Maximum mouth opening	TA-L	0.16	0.71	0.48	0.41	1.97	0.06
		MM-L	0.46	2.23	**0.04** ^*∗*^	0.43	2.07	**0.05** ^*∗*^
		SCM-L	0.47	2.33	**0.03** ^*∗*^	0.41	1.97	0.06
		DA-L	0.50	2.54	**0.02** ^*∗*^	0.44	2.15	**0.05** ^*∗*^

Right axial length	Rest	TA-R	−0.11	−0.48	0.64	−0.15	−0.68	0.51
		MM-R	0.14	0.60	0.56	0.38	1.80	0.09
		SCM-R	0.15	0.67	0.51	0.14	0.62	0.54
		DA-R	−0.47	−2.32	**0.03** ^*∗*^	−0.30	−1.39	0.18
	Clenching in the intercuspal position	TA-R	0.31	1.43	0.17	0.33	1.52	0.14
		MM-R	0.00	0.02	0.99	−0.22	−0.98	0.34
		SCM-R	0.08	0.34	0.74	0.09	0.38	0.71
		DA-R	−0.21	−0.92	0.37	−0.27	−1.21	0.24
	Clenching on dental cotton rollers	TA-R	0.11	0.47	0.65	0.02	0.08	0.94
		MM-R	0.02	0.09	0.93	−0.09	−0.40	0.69
		SCM-R	0.09	0.39	0.70	−0.08	−0.33	0.74
		DA-R	−0.11	−0.48	0.63	−0.05	−0.22	0.83
	Maximum mouth opening	TA-R	0.26	1.19	0.25	0.44	2.16	**0.04** ^*∗*^
		MM-R	0.54	2.78	**0.01** ^*∗*^	0.45	2.17	**0.04** ^*∗*^
		SCM-R	0.40	1.89	0.07	0.38	1.81	0.08
		DA-R	0.67	3.96	**0.001** ^*∗*^	0.68	4.02	**<0.001** ^*∗*^

## Data Availability

The datasets generated and/or analyzed during the current study are available from the corresponding author on reasonable request.
